# Ailing Empires: Medicine, Science and Imperialism

**DOI:** 10.1093/shm/hkag043

**Published:** 2026-05-28

**Authors:** Sam Goodman, Sarah Arens

**Keywords:** medical history, medical humanities, colonial medicine, imperial fragility, global health governance

## Abstract

This special issue explores the entanglements of medicine, science, and imperialism across the nineteenth and twentieth centuries, foregrounding the paradox of ‘ailing empires’ in which health both sustained and destabilised colonial rule. Drawing on case studies spanning South Asia, the Middle East, East Asia, North Africa, and the Mediterranean, the contributions examine how medical knowledge circulated across imperial and transnational networks, shaping governance, social relations, and epistemic hierarchies. The issue highlights medicine as a technology of control and a site of contestation, attentive to questions of race, gender, and class, as well as to the uneven production and validation of scientific authority. Meanwhile, it emphasises the fragility of imperialism, revealing how disease, crisis, and competition exposed the limits of power. The issue underscores the enduring legacies of imperial medicine in shaping modern inequalities and debates over public health and legitimacy.

## Introduction: ailing empires: medicine, science and imperialism

In January of 1941, amidst the dark early years of the Second World War, the British Empire was not well, and neither was Lieutenant Walker of the Scots Guards, then currently in residence on the Highland Chieftain and sailing to Mumbai, or Bombay as he knew it. Writing in the diary he would keep for the next year of his Indian service, Walker noted that he had a fever of 100 degrees, something exacerbated and further spread by the cramped conditions and the piling of the hammocks ‘virtually on top of each other’, leading to the gradual spread of illness and ‘yet more on sick parade’.^1^ Reading Walker’s diary, it soon becomes apparent that the health of the individual and, by extension, the health of the wider British Empire, was a constant concern of both the author and the army in which he served. Over the course of the six-week voyage and many months in various locations across India that followed, sickness and how best to avoid it was never far from Walker’s mind, and all become frequent topics for his pen. For instance, ahead of a scheduled stop at Durban, he writes of how the men were given advice on all matters of personal hygiene, conduct and behaviour, being warned of the depredations of drink, sexual relations with women, and of revealing information lest it be used by the enemy.^2^ Later, in India, he would record of bouts of ‘Bangalore Belly’, worries over whether he has sandfly fever or malaria, and is frequently found drinking (though always in moderation) to while away lazy days off duty.

Walker’s writing suggests an innate understanding of his responsibilities towards his health and, therefore, to the ongoing success of the Empire, with his final lines leaving little doubt as to their connection in his mind: ‘In conclusion, the officer in the Indian Army is bearing upon his shoulders the heritage and past record that the 150 years of British rule has given, and must therefore live up to it, a lot which must never be underestimated’.^3^ Walker perceived the weight of this collective responsibility and sought to meet it through his own personal behaviours; for colonial subjects like him, the history, legacy, and futurity of empire was one where good health and good fortune were interlinked.

Accounts like Walker’s allow us to appreciate, too, the experience and significance of (ill) health and medicine within the context of imperialism on both a micro and macro scale. As his conclusion indicates, Walker was the inheritor of decades of metaphorical and practical conflation between the body of the individual and the body of the state as part of British colonialism, especially perhaps in India where Europeans were always in the extreme minority and where the retention of capable soldiers and administrators mattered enormously to both military and civil establishments alike, but which was common across numerous British colonial possessions in Africa and South East Asia and across other European colonial empires. While it is important to note that the experiences of empire, colonial governance, and medicine of course varied drastically across regions with a history of European colonialism for both occupier and occupied, as well as for the inhabitants of ‘other’ imperial formations, such as the Ottoman Empire and Qing-dynasty China, this special issue seeks to uncover how medical ideas, practices, and conventions circulated transnationally and, frequently, transimperially, across colonial borders to produce shared logics of health, governance, and bodily discipline that were never confined to a single empire or place. By foregrounding the central, yet often unstable, role of medicine in shaping imperial power, the special issue reframes medicine not as a subsidiary concern of imperial history, but as a tool of governance, a site of negotiation, and a lens through which imperial fragilities became both visible and actionable.

As historians such as E. M. Collingham, Pratik Chakrabarti, Sokhieng Au, and Mark Harrison have illustrated, an understanding of medical history of imperialism is crucial to appreciating how medicine and health dominated all aspects of colonial life, from the character and organisation of domestic, personal spaces through to the direction of colonial governments.^4^ Arguably, following Collingham, Harrison and numerous others, there is no imperial history without appreciation of the medical contexts that underpin so many of its key events, decisions or periods. Concerns of health and illness, as well as their intersections with colonialism, are multiple and various, whether in the form of diseases and conditions either endemic to or associated with colonial territories (‘Asiatic’ cholera, for example), the mitigation of environmental factors such as excessive heat and sunlight, or the complexities of establishing and maintaining modern sanitation and medical practice in the face of geographic isolation, a lack of resources, and overwhelming need.^5^ Illness within the context of Empire can therefore appear deeply multifaceted, not least because of the combination of its quotidian and commonplace nature alongside its often deadly and debilitating effects. Whilst Walker’s account represents an empire during a period of acute crisis, the longer history of the connection between medicine and imperialism must be understood, within the frame of the prevailing metaphors of illness of the time, as much more of a chronic condition.^6^

This prevalence and recurrence of illness in colonial spaces resulted in an entanglement of medical knowledge and (imperial) state power, alongside the constant spectre of imperial fragility that existed from the early nineteenth to mid-twentieth centuries, as Nancy Rose Hunt demonstrated in the case of colonial Congo in her seminal work, *A Nervous State* (2015).^7^ On the one hand, the interventionist approach of the British Empire signalled its deep concern for the preservation of public health either as a general social good, as an economic necessity or to serve the more loosely defined but nonetheless important self-justificatory notion of ‘imperial uplift’ and progress made in colonised spaces used to defend the continued British presence. Moreover, the British government’s actions in the provision of medical care demonstrated variously the extent of its resources, its scientific prowess and its continued benevolence, or its ability to control and coerce individuals and groups, all serving to impress its power upon its citizens in complementary ways.^8^ However, concomitant to the exercise of this power is the implied fragility, and possibilities for resistance, that underpin it.^9^ The exposure of European bodies to the various diseases and ailments of colonised territories was a constant cause for anxiety for imperial administrations and communities, and not without good reason. Even in the twentieth century and towards the end of colonial India, the attrition rate to disease for British soldiers and administrators was startling, and serious illness was a common occurrence. For instance, as Walker was enjoying his days by the pool in Poona, the Resident of Baroda in Western India, Cyril Hancock, was dealing with an outbreak of ophthalmia in which his own son was affected; Hancock’s attitude though was to adopt a sense of stoicism he thought necessary or expected of his class, and, writing in his memoir, he states rather blandly ‘price of Empire was a high one which so many of our generation were called to bear’.^10^ Behind this façade lay a very real understanding of the implications if this ‘price’ ever grew too high. However, as Jhana Gourlay argues, the greater the number of healthy Europeans there were to maintain order, the tighter the colonial grip on a country; the fewer, and the edifice of colonial power might cease to look quite so imposing.^11^ The associated metaphors of illness and the Victorian spectre of imperial decline are closely interlinked here. A state beset by disease or mired in crises of public health is vulnerable, and an ‘ailing empire’ is one at risk of a decline from which it might never recover. In times of additional crisis, such as the Second World War where we began with Lieutenant Walker, the duty (or the ‘lot’ as Walker put it) of those in service of European empires like Hancock and millions of others was to maintain the health of the state with paramount importance, lest an ailing empire soon look like one fit only for the grave.

## Ailing empires: critical conceptions

The beginnings of the idea for this special issue lay in conversations on how illness suffused the colonial experience and how the British state variously ‘used’ medicine as an extension of state power. Reflection on these considerations exposed the paradox of imperial control and exercise of power through various means of interventionist efforts in directing public health and behaviour, whether through law, policy or other more subtly coercive means that simultaneously signalled a deep-rooted anxiety over health and an acknowledgement that this power was indeed only ever a few heartbeats away from ruin. Developing and broadening this idea, the goal of the special issue was to further consider the entanglement of medical knowledge and the expression of state power alongside imperial fragility in wider colonial contexts, allowing for comparative analysis of imperial behaviours in contemporaneous empires across the nineteenth and twentieth centuries.

The final work that has resulted from these discussions displays how the intertwined concepts of power and fragility play out across differing temporal, geographical and social contexts, contributing greatly to our knowledge of each contributor's chosen periods and illustrating how imperial medical anxieties affected the Middle East, South Asia, East Asia, North Africa, and the Mediterranean, as well as Europe. Our contributors and their articles explore variously how medicine is repeatedly and sophisticatedly employed as a technology of imperial control, demarcating and inscribing border zones and boundaries of illness and wellness; how empires enable, or move to disrupt, the circulation of knowledge as well as popular, vernacular, photographic and scientific discourses, and how connections are enabled through new kinds of technology and publishing cultures; how the contestation of knowledge and medical debates act as proxies for political tension and other contests of dominance; and how imperial health interventions so often bring a structuring logic to notions of class, racialisation and gender in colonial spaces. It is to our delight that the articles that arose from our original concept and conversations have considered these points in such varied and surprising contexts, but, moreover, have taken the themes of the issue in diverse and fruitful new directions, too. Whether this is, for instance, identity and beautification within domestic spaces, negotiating masculinity, identity and self-image within the burgeoning homosocial spaces of the physical culture movement, or vying for the self-image of a nation across boardroom tables over the restriction of the flow of narcotics, the articles that comprise this special issue bring a productive conceptual and geographic breadth to the analysis of imperial medical history. Given the extent and breadth of its influence both geographic and geopolitical, it is perhaps inevitable that the British Empire remains a frequent presence in many of these articles. Even so, the articles that comprise this special issue offer new insights and perspectives from marginalised territories or subcultural groups within the British Empire’s borders, experiences as important a part of the imperial story as the dominant and more familiar narratives of that colonial history. Similarly, the temporal span of these articles, stretching from the 1810s to the 1950s, offers the special issue further depth, especially in its focus on periods outside of the traditionally understood ‘high’ points of British or French imperial history or those times of distinct crisis. Thus, we might view the weakening authority of an ailing empire, such as the British, in the first half of the twentieth century not only as a diminishment, as so many of its adherents feared, but rather, for our purposes and for those of the communities or individuals in these articles, a productive space of radical new health practices and revealing scholarship alike.

## Scientific authority, transnational circulation, and the fracturing of empire

Sarah Irving’s article examines the precarious position of Arab doctors within the hierarchies of interwar international medical science through the careers of Jamil Tutunji in Transjordan and Tawfiq Canaan in Mandatory Palestine. Centring on disputes over priority, citation, and recognition in European medical journals, the article demonstrates how colonial power relations shaped not only the practice of medicine but also the circulation and validation of scientific knowledge. Tutunji’s frustrated attempts to secure acknowledgement for his early advocacy of sulphur injections in the treatment of ‘general paralysis of the insane’, alongside Canaan’s marginalisation in debates on leishmaniasis, reveal the structural barriers faced by Indigenous practitioners seeking inclusion within Euro-American scientific networks. Despite their education, linguistic competence, and professional alignment with dominant biomedical paradigms, both men encountered scepticism, erasure, or conditional recognition, underscoring the limits of imperial narratives of ‘uplift’ and meritocratic science. Irving situates these experiences within the distinctive political ecology of the British Mandates, where medicine intersected with racialised colonial governance and, in Palestine, with Zionist projects that framed Arab populations as unhygienic, backward, and incapable of self-management. Canaan’s work on leishmaniasis is particularly significant in this regard, as it challenged prevailing assumptions that linked disease to Indigenous practices and instead implicated European military, economic, and infrastructural expansion in its spread. The article traces how medical research in the late Ottoman and Mandate Levant was shaped by transnational networks, missionary institutions, and regional medical schools, yet increasingly fragmented along national and communal lines after the First World War. By foregrounding citation practices as a site of power, Irving shows how scientific authority was unevenly distributed and how colonial and racial hierarchies operated within ostensibly ‘universalist’ disciplines. The article thus contributes to a growing historiography that decentres metropolitan actors and highlights the contested, political nature of medical knowledge production in imperial contexts. In doing so, it offers a compelling account of how ‘ailing’ imperial formations generated not only new diseases and therapies, but also profound epistemic exclusions that structured whose knowledge could count as science.

In ‘Ailing Empires: The Morphine Issue in Sino-foreign Relations at the Dawn of the Twentieth Century,’ Yun Huang examines morphine as a crucial but underexplored problem in Sino-foreign relations during the final years of the Qing dynasty. Moving beyond national or single-empire perspectives, the article demonstrates how morphine (as an industrial pharmaceutical developed and traded by colonial powers) became a focal point of international drug diplomacy, revealing both the vulnerabilities of imperial governance and the contested nature of medical authority at the turn of the twentieth century. Huang traces the morphine issue through three interlinked diplomatic arenas: the revision of commercial treaties between China and Western powers between 1902 and 1904; renewed negotiations over morphine import control between 1906 and 1908; and debates at the Shanghai Opium Commission of 1909. Drawing on Chinese- and English-language sources, including official correspondence, customs records, newspapers, and missionary publications, the article shows how Chinese officials sought to regulate morphine imports as part of broader efforts to assert sovereignty, protect public health, and stabilise state finances in the aftermath of military defeat and internal crisis. These efforts, however, were repeatedly constrained by extraterritoriality, commercial treaty obligations, and the competing interests of colonial powers.

The article situates these diplomatic struggles within a wider medical and imperial context. Morphine’s spread in China was closely linked to Western medical missions, pharmaceutical commerce, and anti-opium campaigns, which together promoted the drug as a modern remedy even as concerns about addiction and misuse grew globally. British and American domestic debates over morphine regulation shaped their diplomatic positions abroad, while missionaries, merchants, and medical professionals acted as key intermediaries in the drug’s circulation. The result was a paradoxical situation in which empires that increasingly recognised morphine as a danger at home continued to profit from or tolerate its spread overseas. By reconstructing the negotiations that culminated in China’s limited success in restricting morphine imports and securing its inclusion in the Shanghai Opium Commission’s resolutions, Huang argues that morphine diplomacy exposes the ‘ailing’ condition of empires themselves. The article thus offers a compelling medical history of imperial decline, highlighting how transnational circulations of drugs, knowledge, and regulation both reflected and reshaped the balance of power in early twentieth-century Asia.

## Bodies, gender, and the domestic frontiers of empire

Mobeen Hussain’s article reconstructs a largely neglected archive of Urdu domestic health manuals written for women in late nineteenth- and early twentieth-century North India, situating them at the intersection of medicine, gender reform, and imperial governance. Focusing primarily on *Tabib al-Nisa* (1934) and *Sinf-i-Nazuk* (1938), both authored by male *hakims* trained in Unani Tibb, the article argues that these texts functioned as key mediators between colonially sanctioned biomedical ideas and locally rooted medical, religious, and ethical frameworks. Far from being marginal or derivative, Urdu manuals articulated hybrid forms of knowledge that reframed women’s bodies as sites of hygienic discipline, reproductive optimisation, and moral responsibility. Addressing an increasingly self-conscious Muslim *ashraf* middle class, they positioned women as primary custodians of familial and, by extension, communal and national health, embedding domestic practice within wider debates on public health, eugenics, and modernity. Hussain demonstrates how these manuals translated the principles of Unani medicine, and particularly its emphasis on balance, regimen, and lifestyle, into accessible, prescriptive advice on menstruation, maternity, exercise, diet, sanitation, and ‘beautification’. In doing so, they responded to colonial anxieties surrounding epidemic disease, sanitation, and general fitness, while also negotiating critiques of *purdah*, *zenana* spaces, and female ‘idleness’. The article further situates these texts within a shifting print culture that privileged didactic, vernacular instruction and increasingly scientific idioms, marking a departure from earlier dialogic or literary forms of women’s advice literature. By comparing male-authored manuals with Sultan Jahan Begam of Bhopal’s *Hifz-i-Sihhat* (1916), Hussain highlights both continuities and divergences in how female health was framed, including the growing authority of scientific language and the gendered politics of authorship. Overall, the article foregrounds domestic health manuals as active sites of knowledge production that illuminate how imperial medicine was vernacularised, contested, and reworked within everyday practices, offering a nuanced account of how women’s bodies became central to late colonial projects of reform, governance, and future-oriented national imagining.

Namrata Ganneri’s article, ‘Crafting New “Health and Beauty” Ideals for Men: Photography and the “Physical Culture Movement” in Late Colonial India’ similarly focuses on the significance of material and publishing cultures within the circulation of health and medical knowledge in late-colonial India and beyond. In a fascinating parallel to Hussain’s article, Ganneri offers an exploration of how global bodily ideals exemplifying a ‘visible muscularity’ that first came to prominence in Europe and the US appealed to and became entrenched among men in late colonial India from the 1930s onwards. Ganneri’s argument is that the bodybuilding aesthetic of European pioneers such as Eugene Sandow, Charles Atlas and George Hackenschmidt, established and popularised by the late nineteenth-century growth in publishing and print cultures, took hold amongst Indian men in urban centres such as Bombay in pre-war, and post-war newly independent India. Moreover, the growth in this male beauty ideal drove the consolidation of a new kind of physical culture and widespread interest in recreational fitness across classes and castes in urban Indian contexts that supplanted more traditionally Indian sports such as wrestling in favour of a Euro-centric model. Ganneri argues that far from an imposition, Indian entrepreneurs cultivated this growth, laying the foundations of the industry of modern commercialised fitness in India around gyms, clubs and other associations.

Beyond the environs of these new spaces of male association though, Ganneri’s position is that such developments transfigured ideas of male masculine beauty in India more generally, offering a means of combatting internalised colonial assertions of the supposed femininity of the Indian male, as well as a corrective to the wasting menial labour of industrial capitalism that had transformed India’s urban landscapes over the preceding decades. As Ganneri outlines, the key to this transformation was also in the ready availability and distribution of photographic illustration of these new body ideals; body image and strength came first to be seen and shared amongst Indian men, and then to be fetishised and desired, not explicitly sexually but predominantly as the physical realisation of the ideal self. The prospect of self-fashioning, and the remaking the Indian male into a newly (re)empowered form has enormous resonance in relation to the growing contemporary clamour for, and eventual realisation of, Indian Independence that would later follow. Such relationships are a further articulation of the connection between the health of the individual and that of the state, with the doctrine of bodybuilding offering a sense of control, strength and discipline that would first transform the Indian individual, and, potentially, then the Indian nation.

Ultimately, however, Ganneri indicates how, for all its potential positives, the healthy body ideal of early twentieth century physical culture was the product and entrenchment of capitalist thinking. Indeed, the commercialisation of the bodybuilding phenomenon by individuals such as Tehmurasp Hormusji Sarkari built on ideas of efficiency and optimisation; such hallmarks of industrialisation underpinned medical practice and became central to the development of what we might now consider a wider ‘wellness culture’ of the period; as Ganneri states ‘optimal physical and mental performance were seen as the ultimate goals of healthy bodies’ and commercialised physicality, aided by the proliferation of photography and visual culture, would be instrumental in the effort to sell the pursuit of this goal to a mass and mainstream audience.

## Empires under threat: quarantine, militarisation, and public health crisis

The final two articles in the special issue transport our attention from South Asia to imperial territories closer to Europe, namely the Ionian Isles and North Africa. Aggelis Zarakostas’ article focuses on the former of these in the context of plague outbreaks across Malta and the wider Mediterranean in the early nineteenth century and considers the actions of Malta's then governor, Thomas Maitland, to limit the spread of contagion. Zarakostas’ article analyses, as he puts it, ‘a rather peculiar episode’ in the history of medical thought where two opposing schools of medical practitioners clashed over their beliefs in the transmission, and by extension, the containment, of plague and other diseases in a series of parliamentary enquires brought between 1819 and 1824.

Zarakostas describes how Maitland was a fierce advocate of the Venetian system of the *lazaretto*, a system of quarantine where those suspected of disease were isolated to specific islands or spaces, and believed such diseases were transmitted by contact. These beliefs brought him into conflict with those who did not share his views, believing instead that infection was spread largely due to atmospheric conditions. Through strict application of systems of segregation and separation modelled on the Venetian systems and reinforced by British military power, however, Maitland’s measures to control the outbreaks of plague were seemingly successful, as he later established before the Select Committee.

Zarakostas’ analysis, however, takes the implications of British medical and quarantine practice much further than the confines of a Select Committee boardroom. The political and imperial context surrounding both the outbreaks and enquiries, as Zarakostas outlines in detail, was as yet not entirely secure or certain, which directed British actions towards a bid to consolidate its hold over its territory. Although the British Navy had maintained an extensive presence in the Mediterranean for many years, Malta had only been in British possession since the Treaty of Paris of 1814 (having earlier become a protectorate of Britain in 1800 after the Maltese expelled the French occupying forces), and the Ionian Isles only ceded to British control after the end of the Napoleonic Wars in 1815. The epidemic outbreak of plague thus became, as Zarakostas illustrates, a means of justifying military rule over the Ionian Isles using the pretence of disease control. The article, and Maitland’s actions, demonstrates how imperial powers might use the state of emergency threatened by illness to substantiate themselves in place, and exercise power over their subjects in the process.

In describing Maitland’s hybrid approach, Zarakostas’ article suggests to us that far from the medical and scientific authority that the British Empire would later go on to portray itself as representing, in many cases such as in the Ionian Isles, British authorities ‘on the spot’ took a more iterative approach to medical practice, utilising and adapting methods and approaches that existed before their arrival and which offered a suggestion of efficacy. The approach to the Ionian Isles is a glimpse of a British Empire, if not in its infancy in these matters, still in a period of development and uncertainty. The legacy of Maitland’s actions, namely his adoption of an established system coupled with modern British military or governmental means, was long lasting and would go on to instruct British actions in numerous other colonial spaces in decades to come.

Continuing the theme of disease control albeit a century hence, Benoît Pouget’s article considers the efforts of Fernand Visbeq’s mission to French Algeria in 1919 and explores how his intervention formed part of a growing militarisation of hygiene and public health in the aftermath of the First World War. In the year following the end of hostilities in Europe, the French military experienced a major outbreak of typhus both within those armies still situated on the Western Front as well as in their North African territories. Alarmed by the potential devastation of such an outbreak and the effect on France’s already depleted military, not to mention the fear of the further circulation of the disease through colonial trade routes, Visbeq was directed by the Under Secretary of State for Military Health to report on disease control methods in the French army in Algeria and charged with bringing the situation under control. As Pouget outlines with reference to Visbeq’s publications, reports, correspondence and field notes, his intervention took the form of a near-ninety day mission to Algeria in April-July 1919 in which he sought to reorganise disease control methods and prophylactic approaches on Pasteurian lines, combining innovations in bacteriology, biomedicine and modern techniques of disinfection and disinfestation alongside older sanitary measures based on isolation. Pouget’s chapter argues that Visbeq’s approach is indicative of the flexibility inherent to Pasteurian methods of the time, as well as offering insight into a nation and a period of broader transition; Visbeq’s hybrid approach of traditional methods backed by the rigour of ‘scientific militarism’, Pouget suggests, is part of a new chapter of biomedicine and biopolitics in the opening decades of the twentieth century that would prove lastingly influential.

Moreover, the article is a reminder that even in the later years of imperialism and in the context of threat to the power of the state, the colonial fringes could be the site of the making of powerful myths and individual fortunes, as well as the spaces in which innovation and new methodologies might gain ground. Visbeq’s narrative of his medical ministrations across the deserts of Algeria is not without a note of deliberate and conscious romance, affirming the trope of the lone, visionary medical practitioner, valorising the breakthrough that comes about because of the decisive individual and their willingness to push back against entrenched medical orthodoxy. In considering Pouget’s article, we might read in the actions of Fernand Visbeq something about the continued importance of the individual in an industrial age, despite the impersonal ‘scientific militarism’ he personally championed; Visbeq’s narrative is a refutation of the brutality and the impersonality of the First World War, whose battles did so much to disabuse European society of the value of personal heroism in the face of impersonal conflict. At the same time as Visbeq’s approach and their meaning are very much of his time, the echoes of the British actions in the Mediterranean a century earlier outlined in Zarakostas’ article are nonetheless clear. The medical measures the British and French enact to control plague and typhus are done so with the full support and authority of the military in both instances, and both Maitland and Visbeq represent the exercise of that imperial power in their respective colonial spaces. Their justification for these actions, delivered through military and governmental channels similarly connect both narratives, as well as further strengthens the interdisciplinary nature of the collection. Both articles exist as discrete medical histories of under considered episodes and places, but they also ask questions of the form, transmission and reception of those histories too. Along similar lines to Maitland, Visbeq’s Mission (with all the linguistic resonance of such a term when applied to colonial and specifically non-Christian spaces) is one that is wholly modern in some senses—rejecting assumptions about disease and instead instituting a methodology rooted in the innovative biomedical practice of the period—yet, at the same time, draws on more traditional tropes, suggesting that the answer to what ails the modern colonial empire might yet be found in the heritage and past record of its preceding centuries.

## New directions in the history of medicine and empire

This special issue thus intervenes in an active and evolving historiography that has foregrounded medicine and public health as central to the making, maintenance, and contestation of imperial power. Foundational works such as the aforementioned Pratik Chakrabarti’s *Medicine and Empire*, which traces the intertwined trajectories of Western medicine and imperial expansion across continents, have established the mutually constitutive histories of empire and medical authority. More recent scholarship, including *A Cultural History of Medicine in the Age of Empire*, edited by Jonathan Reinarz (2023), underscores how medicine, technologies, institutions, and populations circulated transnationally, shaping not only professional cultures but also everyday encounters with health and illness.^12^ Meanwhile, studies such as Jim Downs’s *Maladies of Empire* (2021) reveal how the violence and epistemic exclusions of colonialism were embedded in the production of modern medical knowledge itself, illustrating the racialised context in which epidemiology and public health programmes developed.^13^ Building on interventions like these, the contributions in this issue extend existing studies in three key ways. First, by integrating cross-imperial comparison: from the British Mandates in the Middle East to Qing/foreign treaty negotiations in China, and to Ottoman, French, and Mediterranean contexts, the special issue emphasises that scientific authority and medical regulation were not bound by single empires but circulated across overlapping jurisdictions and contested global networks of practice and diplomacy. Second, several articles spotlight lesser-studied actors, regions, periods and media, including vernacular Urdu health manuals, colonial photography, and medical citation practices. In doing so, Hussain, Ganneri, and Irving broaden the archive of imperial medicine beyond metropolitan publications and institutions. Similarly, Zarakostas, Pouget and others avoid analyses of imperialism in apogee or terminus, offering us insight into periods of development that would lead to future consolidation or episodes of imperial retrenchment that go beyond typical approaches in the field. Third, the issue highlights how gendered and racialised biopolitics shaped the governance of bodies and populations, showing how empire confronted its own fragility through medical interventions that both enforced and exposed limits of authority. In doing so, ‘Ailing Empires’ contributes to global histories of public health that view medicine as a fluid, contested, and always-already politicised field of knowledge—one in which the very idea of ‘health’ was woven into projects of governance, violence, and negotiation across imperial worlds.

## Conclusion: the afterlives of ailing empires

In exploring the medical politics and practices of past imperial states, our hopes have been two-fold; firstly, to consider the historical entanglement of medical knowledge and the expression of state power in a variety of colonial contexts and secondly, to reflect on how their approaches, ideas and legacies might still resonate with us today. In doing so, the context of our own moment, its medical history, and its influence on our approach cannot be ignored. The origins of this collection began in the months just prior to the COVID-19 pandemic, and its articles written, edited and redrafted during its various waves, phases and tiers against a background of state interventions exemplifying the full range of governmental competence, or lack thereof. Though the subject matter and the context were of course greatly different, the echo of many of the same concerns our authors explore in these articles was hard to ignore, especially scientific and political debates over cause and treatment of communicable disease, the efficacy or otherwise of disease control, the return of widespread measures of quarantine, and, of course, the implications of disease on individual behaviours, habits and identity and concepts of self-regulation in relation to health. Though divorced from European empires by time and political change in the intervening decades, the acute legacies of colonial public health measures were suddenly made visible. For instance, the UN Committee on the Elimination of Racial Discrimination (CERD) attributed vaccine hesitancy among people of African and Asian descent to the historical effects of colonialism; such reluctance was especially visible in places such as the Democratic Republic of Congo where a lack of public vaccine take-up had a clear historical root in the actions of the former Belgian colonial government, but similar fears over a lack of trust in state public health intervention were expressed in the UK, France and the US with reference to diasporic communities from formerly colonised countries.^14^ The COVID-19 pandemic also coincided with renewed instability in global health governance, not least through the initiation of a formal withdrawal of the US from a range of international health initiatives during the first Trump presidency, which significantly altered the landscape of global medical coordination. This raises further questions about what becomes of the infrastructures, hierarchies, and dependencies of imperial and post-imperial health systems when such dominant actors abruptly withdraw from them, and how their absence reshapes both capacity and legitimacy in global health provision.

Beyond their emergency contexts, however, such questions point us towards consideration of the persistence of health inequalities shaped by the continuation of imperial medical structures and, as the UN indicates, the hierarchies of knowledge, power, and identity that inform them. For all of their specificity in describing their own time, place and events, the articles that comprise this special issue and their themes remain relevant to us and to the topography of the landscape of contemporary care, whether that involves how to approach the control and classification of narcotic substances, the changing nature of wellness advice and the circulation of medical knowledge within contemporary media and publishing cultures, and how these have become primary arenas of online mis- and disinformation, or even how an ailing health service might reflect on the state responsible for its running. Moreover, and to bring us back to Lieutenant Walker and the opening of this piece, the articles invite us to reflect on our own understanding of the relationship between health and the individual, considering, perhaps, to what extent those ideas we hold are similarly a product of circumstance as much as medical science.

## Footnotes

1 Second World War diary of Lieutenant Walker, of the Royal Scots Regiment, British Library, India Office Records, Acc.13462, 1940–41, npag.

2 Diary of Lieutenant Walker, npag.

3 Diary of Lieutenant Walker, npag.

4 See: Sokhieng Au, *Mixed Medicines: Health and Culture in French Colonial Cambodia* (Chicago: University of Chicago Press, 2011); E. M. Collingham, *Imperial Bodies: The Physical Experience of the Raj 1800–1947* (London: Polity Press, 2001); Pratik Chakrabarti, *Medicine and Empire1600–1960* (Houndmills: Palgrave Macmillan, 2014); Mark Harrison, *Climates and Constitutions: Health, Race, Environment and British Imperialism in India 1600–1850* (Oxford: Oxford University Press, 1999).

5 The academic literature available on the history of imperialism and medicine is vast, and the following list is illustrative and by no means exhaustive: David Arnold, *Colonizing the Body: State Medicine and Epidemic Disease in Nineteenth Century India* (Berkeley: University of California Press, 1993); Anna Crozier, *Practising Colonial Medicine: The Colonial Medical Service in British East Africa* (London: I B Tauris, 2007); Roy McLeod and Milton Lewis, eds, *Disease, Medicine and Empire: Perspectives on Western Medicine and the Experience of European Expansion*, 2nd ed. (London: Routledge, 2022); Delphine Peiretti-Courtis, *Corps noirs et médecins blancs : La fabrique du préjugé racial, XIXe-XXe siècles* (Paris: La Découverte, 2021); Suman Seth, *Difference and Disease: Medicine, Race, and the Eighteenth-Century British Empire* (Cambridge: Cambridge University Press, 2018); Aro Velmet, *Pasteur’s Empire: Bacteriology and Politics in France, Its Colonies, and the World* (Oxford: Oxford University Press, 2020).

6 Despite their potentially problematic usage, especially where they might serve to detract from the lived reality of illness, medical metaphors remain a potent and perennial literary and cultural presence, reflecting their use and utility in communicating meaning in a range of temporal and political contexts or as a form of advocacy in relation to certain movements. See: Jackie Stacy, *Tetratologies: A Cultural Study of Cancer* (London: Routledge, 1997); Lorenzo Servitje, *Medicine is War: The Martial Metaphor in Victorian Literature and Culture* (New York: SUNY Press, 2021).

7 Nancy Rose Hunt, *A Nervous State: Violence, Remedies and Reverie in Colonial Congo* (Durham: Duke University Press, 2016).

8 Anna Greenwood, writing in *The Lancet*, takes a more positivist approach to imperial medicine, arguing that the extent of resources committed to health care in colonial spaces can be seen as one of the more ‘defensible imperial beneficences’. See Anna Greenwood, ‘Diagnosing the Medical History of British Imperialism’, *The Lancet*, 400 (2022), 726–27.

9 It is therefore misleading to frame colonial medical provision in terms of ‘soft power’ (as opposed to military intervention). While it projected scientific authority and humanitarian intent, colonial medicine operated as a form of governance in which care, surveillance, and coercion were closely intertwined, even as it revealed the fragility of imperial authority and its dependence on maintaining legitimacy. For instance, as Delphine Peiretti-Courtis demonstrates in the post-Second World War period, the rise of international health organisations such as the WHO intensified competition over public health in Africa, prompting colonial authorities to reassert their role by emphasising the humanitarian success of their ‘local’ interventions (‘Colonial medicine and “Black strength” in the French colonies of Africa during the long Second World War’, in Marie-Luce Desgrandchamps, Laure Humbert, Bertrand Taithe and Raphaële Balu, eds, *Medical Care, Humanitarianism and Intimacy in the Long Second World War, 1931–1953* [Manchester: Manchester University Press, 2025], 53–83, 60). However, competition highlights, as both Deborah Neill and Guillaume Lachenal argue, the long-standing instability and contested legitimacy of colonial medical authority (see Deborah Neill, *Networks in Tropical Medicine: Internationalism, Colonialism and the Rise of a Medical Speciality, 1890–1930* [Stanford: Stanford University Press, 2012] and Guillaume Lachenal, *Le Médecin qui voulut être roi: sur les traces d’une utopie coloniale* [Paris: Seuil, 2017]).

10 Lt-Col Sir Cyril Percy Hancock (b. 1896), IPS 1920, Resident, Western India States and Baroda 1944–47, ‘Memoirs of his career in the Indian Army and the IPS’, British Library, Mss Eur F226/12: 1914–1944, 9.

11 See: Jhana Gourlay, *Florence Nightingale and the Health of the Raj* (Aldershot: Ashgate Press, 2007), 27.

12 Jonathan Reinarz, ed, *A Cultural History of Medicine in the Age of Empire* (London: Bloomsbury Academic, 2023).

13 Jim Downs, *Maladies of Empire: How Slavery, Imperialism, and War Transformed Medicine* (Cambridge, MA: Belknap Press, 2023). See also Julia Hartley and Sarah Arens, eds, ‘Science and Culture after the Advent of Race’, special issue of *Dix-Neuf*, 28.1 (2024).

14 Sarah Johnson, Covid vaccine inequity due to ‘racism rooted in slavery and colonialism’, *The Guardian*, 30 April 2022, https://www.theguardian.com/global-development/2022/apr/30/covid-vaccine-inequity-due-to-racism-rooted-in-slavery-and-colonialism, Accessed: 5 January 2026.

